# A new parallel pipeline for DNA methylation analysis of long reads datasets

**DOI:** 10.1186/s12859-017-1574-3

**Published:** 2017-03-09

**Authors:** Ricardo Olanda, Mariano Pérez, Juan M. Orduña, Joaquín Tárraga, Joaquín Dopazo

**Affiliations:** 1 Departamento de Informática, Universidad de Valencia, Avda. Universidad, s/n, Burjassot Valencia, Spain; 2 Department of Computational Genomics, Centro de Investigación Príncipe Felipe, Eduardo Primo Yúfera, s/n, Valencia, Spain

**Keywords:** High performance computing, DNA methylation, Parallel pipeline

## Abstract

**Background:**

DNA methylation is an important mechanism of epigenetic regulation in development and disease. New generation sequencers allow genome-wide measurements of the methylation status by reading short stretches of the DNA sequence (Methyl-seq). Several software tools for methylation analysis have been proposed over recent years. However, the current trend is that the new sequencers and the ones expected for an upcoming future yield sequences of increasing length, making these software tools inefficient and obsolete.

**Results:**

In this paper, we propose a new software based on a strategy for methylation analysis of Methyl-seq sequencing data that requires much shorter execution times while yielding a better level of sensitivity, particularly for datasets composed of long reads. This strategy can be exported to other methylation, DNA and RNA analysis tools.

**Conclusions:**

The developed software tool achieves execution times one order of magnitude shorter than the existing tools, while yielding equal sensitivity for short reads and even better sensitivity for long reads.

**Electronic supplementary material:**

The online version of this article (doi:10.1186/s12859-017-1574-3) contains supplementary material, which is available to authorized users.

## Background

The introduction of NGS (Next Generation Sequencing) technologies has made possible the sequencing of genomic DNA in a short time (days), producing billions of short DNA samples (commonly denoted as reads). The typical read length produced by current NGS sequencers ranges from 50 to 400 nucleotides (nts), though new sequencers yielding reads of several thousand nts are already in the market [1]. Also, the upcoming generation of portable high-throughput sequencers is expected to produce huge datasets containing very long reads [2].

A particular topic of DNA analysis is DNA methylation. By aligning and comparing (mapping) bisulfite-treated reads to the genomic DNA sequence, it is possible to determine the methylation of each pair-base. Several methylation tools have been developed over recent years [3–6]), but all of these tools decrease their performance for reads whose length exceeds 100-150 nts. In order to tackle this problem, we developed HPG-Methyl, a new tool for analyzing the methylation of bisulfite reads [7]. The strategy implemented in this tool was the use of a parallel pipeline that aligns the reads by using both the Burrows-Wheeler Transform (BWT) [8] and the Smith-Waterman algorithm (SWA) ([9]). The combination of both algorithms in HPG-Methyl provides the best performance for all the considered methylation analysis tools. Nevertheless, for large datasets with long reads the execution times are still too long, preventing the analysis tasks from being carried out in an interactive way.

In this paper, we propose a new strategy for methylation analysis that greatly reduces the required execution time of the mapping tools while yielding a better level of sensitivity, particularly for datasets composed of long reads. This strategy can be exported not only to other methylation analysis tools, but also to DNA and RNA analysis tools. It consists of two independent techniques: first, we use a bidirectional implementation of the BWT that tries to map each read onto the reference genome simultaneously starting from both read ends (and proceeding to the center of the read), instead of mapping each read from one end to the opposite one.Unlike other implementations of bidirectional BWT [10, 11], it allows up to two EIDs when aligning a read, increasing the performance of the alignment tool. This technique does not add any computational cost to the BWT, and it helps to discard wrong alignments more efficiently. Second, a different pipeline scheme [7] is used for those reads which cannot be completely mapped by using the BWT. The new pipeline scheme merges several stages into a single but more flexible stage, based on the BWT. This new stage provides considerably fewer candidate regions of the genome where each considered read can be mapped, although these regions are much more likely to be correct. As a result, the use of the SWA in the pipeline is greatly reduced, and it maps much shorter read segments. Since the computational cost of the SWA depends on the read length, and these techniques greatly reduce the number and length of the read segments mapped by using the SWA, the proposed strategy greatly improves the performance of the methylation tools, allowing them to linearly scale with the length of the reads. We have implemented this strategy in HPG-Methyl, developing a new version denoted as HPG-Methyl2. The performance evaluation results show that the new tool achieves execution times one order of magnitude shorter than the existing tool, while yielding slightly better sensitivity for short reads and significantly better sensitivity for long reads.

## Implementation

### A new implementation of the Burrows-Wheeler Transform

The Burrows-Wheeler Transform (BWT) is a compression procedure originally designed for data (text) compression [12, 13]. Later, it was used as a backward search method (from the last character of the query string to the first one) to efficiently align short sequencing reads against a large reference sequence such as the human genome, allowing errors (mismatches), insertions or deletions (EIDs) [8]. Many software tools and BWT implementations have been proposed for sequence alignment [14, 15]. Although the computational cost of the BWT increases with the number of allowed EIDs due to the search tree exploration process, most of the existing implementations use pruning or greedy schemes to avoid an exponential cost [8, 10, 15].

**Fig. 1:**
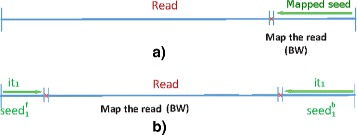
Alignment of a read sequence. Using **a** unidirectional BWT **b** bidirectional BWT

Nevertheless, the BWT is used to perform a unidirectional backward search in all cases. The BWT starts from one end of the read, trying to align as many nucleotides (nts) of the read as possible to a sequence of the reference genome, until an EID is found. Figure 1 a) illustrates the mapping of a portion of the read, denoted as a *segment*. The mapping procedure is as follows: the last nucleotide of the read is searched in the reference genome. Next, the sequence of the last two nts of the read is searched on the reference genome, then the sequence of the last three nts, and so on. In each search, a new nucleotide from the end of the read is added to the sequence to be found. This procedure is repeated, until an EID is reached. At that point, a segment of the read will be aligned to one or more locations of the reference genome. Also, it is possible to use the BWT to perform the alignment in the opposite direction [10] by constructing the Ferragina and Manzini Index [16] for the reversal of the read.

Unlike other implementations of bidirectional BWT [10, 11] our BWT implementation allows up to two EIDs when aligning a read. To be precise, it can be configured to allow 0, 1, or 2 EIDs. As illustrated in Fig. 1 b), the bidirectional alignment of the read simultaneously starts from both read ends, looking for the occurrences of the first (and last) nts. This strategy allows the duplication of the supported EIDs without exponentially increasing the size of the search tree. As in unidirectional implementations, further nts are added to the initial sequences, until too many EIDs are found. The main differences is that when the procedure finishes, we have two mapped segments instead of one, and the distance between the segments can also be used to search the correct mapping of the read in the reference genome. The main difference with other bidirectional implementations is that the proposed one can be configured to allow two EIDs, and this fact can help to increase the performance of the alignment tool, as shown in the “Results” section.

### Implementation in a parallel pipeline

The BWT implementation and use described in the previous section can be used in any alignment process. In order to prove its potential, we have integrated this version of the BWT in the parallel pipeline of a software tool designed for methylation analysis, termed *HPG-Methyl* [7].

Like most of current software tools [8, 14], HPG-Methyl combines multi-seeding with dynamic programming methods such as the SWA. It first uses BWT to align small segments of the reads (seeds) in the genome. Depending on the location of the seeds, one or more candidate areas are considered for aligning the rest of the read segments using SWA. In this way, the higher computational cost of the SWA algorithm is required only for inter-seed spaces, instead of the entire read.

**Fig. 2:**
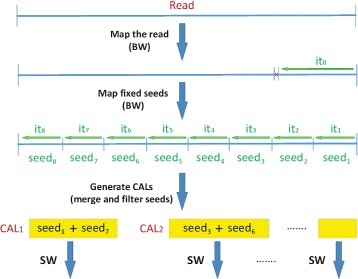
Parallel pipeline in HPG-Methyl tool

Figure 2 illustrates the main processing performed by HPG-Methyl [7] on each read of the input dataset (although some pre and post-processing are needed due to methylation, this processing is carried out in other stages that remain unchanged). For each pre-processed read, the BWT stage tries to align the whole read against the reference genome using the unidirectional BWT. If this is achieved, the read is stored for post-processing. However, the BWT can efficiently handle only a few EIDs, and the probability of alignment failure when using the BWT increases with the read length. When the alignment is not achieved, the next HPG-Methyl stage of the BWT consists of splitting the read into *n* segments denoted as *seeds* (8 segments in the figure), and independently aligning each seed of the read against the reference genome by using the BWT. This stage is called seeding. Each of the seeds can be aligned everywhere, but the alignment of two or more seeds at short distances can reveal candidate areas for read alignment. Thus, the next stage of the pipeline consists of selecting those areas in the reference genome (with lengths similar to the read length) where two or more seeds have been aligned. These areas are called Candidate Alignment Locations (CALs), since the potential alignment of the whole read in these areas need to be carefully analyzed. The next pipeline stage consist of using another more accurate algorithm (the SWA, which requires a much higher computational cost) to align the read against each of the CALs. However, it must be noted that the alignment of the read against the CAL means a huge reduction in the computational cost, compared to the alignment of the read against the whole genome.

In order to take full advantage of the BWT implementation described in the previous section, we have changed the HPG-Methyl pipeline, denoting this new pipeline as HPG-Methyl2. HPG-Methyl2 merges several stages into a single but more flexible stage, based on the BWT. The new stage provides considerably fewer candidate regions of the genome where each considered read can be mapped, although these regions are much more effective. As a result, the use of the SWA in the pipeline is greatly reduced, and it is used to map much shorter read segments.

**Fig. 3:**
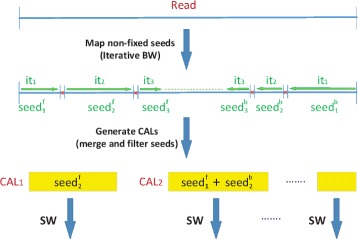
New parallel pipeline in HPG-Methyl2 tool

Figure 3 illustrates the new pipeline. Since the BWT stage now yields the alignment of two read segments, the BWT stage has been modified to include the seeding and CAL search stages. The BWT stage starts by using the BWT to align the whole read. However, in case of failure (when reaching the maximum number of EIDs that BWT can efficiently handle), the bidirectional BWT will yield the alignment of two read segments, the forward and backward segments. These segments can be considered as seeds, and the areas where these segments have been aligned (including the distances between the two segments) will later be considered as potential CALs. Next, the inner limits of the aligned read seeds are annotated (the inner limits are those nts in the read where each iteration finishes because it has found too many EIDs. These limits are illustrated in Fig. 3 by red crosses.), and the inner read segment between these limits is considered again as the read to be aligned. In this way, Fig. 3 shows how the BWT is iteratively applied to align the inner segment of the read that has still not been aligned, until the length of the inner segment is lower than a certain threshold. At that point, we will have *n* seed pairs (forward and backward seeds) that cover most of the read, and they can be merged and filtered to generate new CALs. In effect, we consider that a new CAL is formed by either any seed whose length is greater than a given threshold, or by any group of seeds (two or more) whose alignment is found within a distance lower than the read length (including the length of the seeds). Figure 3 illustrates the cases where the second forward seed ($seed_{2}^{f}$) fulfills the former criterion and the pair formed by the first forward seed ($seed_{1}^{f}$) and the second backward seed ($seed_{2}^{b}$) fulfills the latter.

The next stage in the pipeline is the SW stage, where the SWA is used to study the alignments of the read in the CALs yielded by this stage, as in the HPG-Methyl software [7]. However, it must be noted that there is a crucial difference between the HPG-Methyl pipeline and the strategy described here (HPG-Methyl2): in the former case, only one iteration of the BWT was applied, and for those reads not mapped in that iteration the seeding stage generated new and short read seeds from scratch. As a result, the length of the unmapped segments between seeds to be studied using the SWA could be any number between the length of the seed and the length of the read. In the latter case (HPG-Methyl2), the BWT is iteratively applied before generating the CALs, in such a way that the correct CALs will probably contain more than two seeds, and therefore the length of the unmapped segments to be studied using the SWA will be much shorter. In this way, the computational cost of the pipeline remains closer to a linear cost than to quadratic cost with regard to the read length. Also, the existence of more seeds within each CAL helps to align more reads, while the probability of multiple alignments for each CAL decreases. Finally, the probability of generating CALs which cannot be aligned is greatly reduced.

## Results and discussion

In this section, we present a comparative performance evaluation of the BWT implementation and deployment described in the previous section. We have denoted this implementation as *HPG-Methyl2*. For comparison purposes, we have also evaluated two additional tools: *HPG-Methyl* and Bismark. These tools were selected because they yielded the best performance at the time HPG-Methyl was evaluated [7]. We have measured the sensitivity and execution time yielded by the considered software tools when using synthetic as well as real datasets. The former datasets have been extracted from the reference genome (which in turn was downloaded from Ensemble.org (http://grch37.ensembl.org/Homo_sapiens/Info/Index)), while the latter ones have been obtained from the European Nucleotide Archive (http://www.ebi.ac.uk/ena/data/view/SRR309230 and SRR837425). All the datasets contain fixed read lengths, and we have considered datasets of different reads lengths, from 75 to 3200 nts. All the synthetic datasets contain four million reads. The performance evaluation has been carried out on the same computer platform used to evaluate the HPG-Methyl tool [7], a computer based on an Intel i7-3930K processor (http://ark.intel.com/products/63697) with 48 Gbytes of RAM. Nevertheless, the average use of memory shown by all the considered tools did not exceed 17 Gbytes of RAM. We have used the default parameter settings in the execution of the Bismark tool, other than the number of parallel execution threads. The Hpg-Methyl tools is the only tool where the optimum parameter settings are not automatically computed based on each read length, and therefore these parameters should be explicitely used in the command line according to the length of the reads in the dataset, as described in the README.TXT file (see the “Availability of data and materials” section). The HPG-Methyl2 tool automatically computes the optimum parameter settings for each read length, so it only needs the number of parallel threads to be used in the execution. An example execution command for each of the considered tools can be found in the “Additional file 1” (see the Additional file section).

Table 1 shows the sensitivity yielded by the considered tools for synthetic datasets with a mutation rate of 1%. In this table, the columns labeled “R” shows the percentage of reads correctly aligned, and the columns labeled “W” shows the percentage of reads wrongly aligned. The aggregated value of both columns represents the ratio of reads aligned by each software tool. All the software tools have been configured to use the maximum number of threads (one per CPU core). The “–” value means that the executions had not finished after three days (72 h, 4320 min), and they were aborted. This table shows that HPG-Methyl2 yields the greatest sensitivity, although the differences with regard to the other software tools remain constant (about 2-3% in most cases). These results show that HPG-Methyl2 is an efficient tool (in terms of sensitivity) for datasets of any read length even if the mutation rate is high.

**Table 1:** Sensitivities yielded for a synthetic dataset with a mutation rate of 1%

Length	HPG-Methyl2		HPG-Methyl		Bismark	
(nt)	R	W	R	W	R	W
75	95.50	0.69	93.37	0.62	88.30	0.1
150	99.01	0.46	96.87	0.80	94.59	0.08
400	99.75	0.18	97.55	0.48	97.55	0.10
800	99.93	0.06	97.58	0.43	98.45	0.08
1600	99.75	0.06	96.94	0.48	–	–
3200	99.68	0.08	96.42	0.49	–	–

Table 2 shows the execution times for the datasets with a mutation rate of 1%. It shows that HPG-Methyl2 is the fastest tool. For datasets with longer reads it shows a much lower increase in the required execution time than HPG-Methyl, while the time required by Bismark is several orders of magnitude higher.

**Table 2:** Execution times (min.) for processing the synthetic dataset (1% mutation rate)

Length (nt)	HPG-Methyl2	HPG-Methyl	Bismark
75	1.288	1.366	62.579
150	1.550	1.95	106.173
400	5.041	10.85	248.107
800	11.260	50.6	1246,89
1600	34.440	996.567	–
3200	164.593	7733.38	–

Also, we have tested the considered tools with real datasets (SRR309230, SRR837425) containing 16.6 million bisulfite reads coming from *Homo sapiens*. The length of the reads in each file is 75 and 100 nts, respectively. Table 3 shows the percentage of reads mapped. It shows how HPG-Methyl2 again yields the greatest sensitivity for real datasets, although the performance differences are not very significant due to the short read length.

**Table 3:** Sensitivities yielded for real datasets

Dataset	HPG-Methyl2	HPG-Methyl	Bismark
SRR309230_1	88.40	87.71	71.81
SRR837425_1	84.34	82.75	68.42

Finally, Table 4 shows the execution times required to align the real datasets. It can be seen how both versions of the HPG-Methyl software require similar execution times for the SRR309230 dataset, while Bismark yields much longer execution times. The reason for this behavior is that the read length in this dataset is too short for HPG-Methyl2 to significantly increase the performance. However, the performance differences among the considered software tools for the SRR837425 datasets are more significant, the execution time of HPG-Methyl2 being half of that required by the HPG-Methyl tool.

**Table 4:** Execution times (min.) for processing the real datasets

Dataset	HPG-Methyl2	HPG-Methyl	Bismark
SRR309230_1	11.333	12.053	82.120
SRR837425_1	8,404	19.047	95.194

## Conclusions

The performance evaluation results show that the new software tool achieves execution times one order of magnitude shorter for long reads, while yielding equal or better sensitivity. The strategy employed in this software can be exported not only to other methylation analysis tools, but also to DNA and RNA analysis tools. As a future work to be done, we plan to apply the same strategy of BWT deployment to other existing software tools with a similar parallel pipeline, such as the HPG Aligner [17, 18].

## Availability and requirements

**Project name:** HPG-Methyl2**Project home page:** https://github.com/grev-uv/hpg-methyl **Operating System:** The software has been tested on Ubuntu Linux**Programming language:** C**License:** GPL v2

## Additional file

Additional file 1 Text document containing an example of the command launched to execute each of the tools. (TXT 2 kb)

## Abbreviations

BWT: Burrows-wheeler transform
Cs: Cytosines
EIDs: Errors, insertions or deletions
NGS: Next generation sequencing
nts: Nucleotides
PCR: Polymerase chain reaction
SWA: Smith-waterman algorithm
